# Traditional alcohol production and use in three provinces in Vietnam: an ethnographic exploration of health benefits and risks

**DOI:** 10.1186/1471-2458-14-731

**Published:** 2014-07-18

**Authors:** Bich Ngoc Luu, Thi Thieng Nguyen, Ian M Newman

**Affiliations:** 1 Institute for Population and Social Studies, National Economics University, Hanoi, Vietnam; 2 Department of Educational Psychology, University of Nebraska, Lincoln, NE, USA

**Keywords:** Alcohol, Ethnography, Traditional, Non-commercial, Informal, Illicit, Homemade, Moonshine Vietnam

## Abstract

**Background:**

Gaps exist in knowledge about the production and use of traditional alcohols, particularly in Asia. This study adds new information about the nature, production and sale of traditional distilled spirit alcohol in Vietnam.

**Method:**

This was an ethnographic study of traditional distilled spirit alcohol production in rural areas of three provinces in Vietnam. Researchers interviewed more than 300 individuals and recorded responses to general open-ended questions about local alcohol production. Interviews were recorded, transcribed, and studied to discern what information about traditional alcohol was important to the speakers.

**Results:**

Methods of production followed long-held traditions. Participants listed both personal and community benefits (economic, health, and social) from traditional alcohol making. Older people favoured traditional alcohol, while younger people favoured brand-name beer. Typically people consumed 2-4 drinks daily, mainly at meal times. People consumed more alcohol at special events and festivals. Distribution patterns ranged from low-risk distribution to family and neighbours to high-risk distribution by an agent who might combine alcohol from several producers, which increases the opportunity for dilution and adulteration. The most commonly listed health risks associated with locally-made alcohol were local air pollution and water pollution; participants also mentioned traffic crashes and bad public behaviour. Depending on the location, community leaders reported that production may be relatively stable or it may be declining.

**Conclusions:**

Traditional alcohol manufacture, sale, and use in Vietnam is a long-standing practice and low- to moderate-risk to health. There do not appear to be instances of accidental or intentional contamination. Urbanization seems to be affecting the market share of traditional alcohol as urbanized youth turn to branded products, mainly beer, making traditional alcohol making and consumption an activity mainly linked to older people in rural areas. In the rural areas surveyed, significant economic and social benefits are derived from traditional alcohol manufacture, sale, and use. Policy makers designing ways to reduce alcohol-related risks and harms need to give thoughtful consideration to the role traditional alcohol plays in the local society and to suggest changes that do not create unintended problems.

## Background

The World Health Organization (WHO) has outlined global strategies for member states to reduce the harmful use of alcohol [1]. Worldwide, according to the most recent estimate of the WHO, 24.8% of alcohol consumed is unrecorded alcohol [2]. The *Global Strategy* calls for reducing the public health impact of illicit and informally-produced alcohol—these kinds of alcohol are largely unregulated, untaxed, and not officially recorded, and are typically made using traditional methods by individual families or by small village factories whose facilities are often neither registered nor inspected (p. 30) [2]. One of the challenges stated in *Global Strategy* is that of gathering and disseminating information about illicit and informally-produced alcohol, especially where there are still “substantial gaps in knowledge” (p. 7) [1] and especially in developing countries. It is important to know as much as possible about illicit and informally-produced alcohol in community contexts in order that policies to reduce its harm do not contribute to other harms.

Most of what we currently know about illicit and informal alcohol involves descriptions of its related harms and comes from eastern Europe and Africa [3]. The aforementioned “substantial gaps in knowledge” exist about the way informal alcohol is produced, how much is produced, its role in the lives of those who make and consume it, and the role it plays in the social and economic life of the community [4]. This lack of information is most apparent in Asian countries, which possess some of the richest and oldest continuous traditions of alcohol production.

This paper provides information about production, price, distribution patterns, patterns of use, reasons for drinking, social significance of drinking, and health risks of drinking traditional alcohol in rural areas of three provinces. The information was garnered from in-depth interviews with 240 local village residents, 60 members of local Commune Peoples’ Committees and 6 individual interviews with village leaders in rural villages in three provinces of Vietnam.

The decision to conduct an ethnographic study was made because we believed the collection of information from interviews would provide a basis for the development of more formal qualitative approaches that could then lead to better quantitative methods of data collection and the future development of culturally-sensitive surveys of larger samples. Policy development will need both qualitative data and quantitative data to be effective. An ethnographic study aims to capture the meaning of ordinary activities in naturally-occurring settings. The main methods of ethnography are observations, interviews and discussions. The goal is to collect information in a way that minimizes investigator bias [5,6]. Collecting information in an unbiased manner is important to understanding alcohol use in a society where it has not yet been extensively studied. For example, an investigator from a culture with long-standing negative attitudes about alcohol’s moral and health impacts might misunderstand or discount a different culture’s positive statements about alcohol use or might unconsciously amplify negative statements about alcohol’s impact. This study attempted to document information in precisely the way local individuals reported and described their perceptions of the risks and benefits of traditional alcohol.

The WHO’s *Global Strategy* document uses the terms “illicit alcohol” to mean alcohol that is prohibited and the term “informally produced alcohol” to mean alcohol that is not prohibited (p. 15) [1]. Another term commonly used is “non-commercial alcohol”, [3] a term meaning essentially the same as “unrecorded alcohol”, i.e., alcohol that is “not reflected in the official statistics such as production, trade and sales figures” (p. 3) [3] None of these labels is completely accurate for this study, therefore this paper will use the term “traditional alcohol” to mean distilled alcohol that has been made according to centuries-old methods, that predates commercial brands of alcohol, that is made to meet needs and tastes of local people, and for which the society that makes it has long-standing, culturally-ingrained controls on its manufacture and use.

### Estimates of traditional alcohol consumption and alcohol disorders

In 2004, the WHO reported the annual per capita consumption of alcohol among people 15 years and older in Vietnam for the years 2000/2001 to be 1.35 litres of recorded pure alcohol per year [7], and the consumption of unrecorded alcohol was reported to be 1 litre per year [8]. In 2011 WHO reported the annual per capita consumption of pure alcohol for the years 2003/2005 at 1.1 litres of recorded pure alcohol and 2.7 litres of unrecorded alcohol per year [9]. In 2014 WHO estimated annual per capita consumption at 5.1 litres of pure alcohol of which an estimated 1.7 litres was unrecorded alcohol [2]. There were no explanations offered for the differences in estimates for the 2004, 2011, and 2014 reports. The estimate of per capita consumption of unrecorded alcohol was based on “empirical investigations and expert judgments” and an algorithm (page 283, table IV.IV) [10]. The unrecorded alcohol included traditional alcohol and alcohols carried across national borders, legally or illegally, and alcohol produced for commercial/industrial use that was incorporated in beverage alcohol (p. 5 Box 5) [10].

In the 2014 report, WHO estimated that in Vietnam 8.9% of males and 0.9% of females have an alcohol use disorder (AUD) and that 5.9% of males and 0.1% of females are alcohol dependent [2].

Problems of defining, documenting and quantifying use of traditional alcohol around the world are described in: *Moonshine Markets*[11], which describes the production and use of traditional alcohol in several parts of the world and discusses policy related issues; *Producers, Sellers and Drinkers: Studies of Non-commercial Alcohol in Nine Countries*[9], a report on non-commercial alcohol production in Sub-Saharan Africa [12], central and eastern Europe [13], and Sri Lanka [14], and a variety of reports from other countries, mostly about production and use in Africa and eastern Europe [15-21]. Recently Rehm and colleagues have published a review of the epidemiology, consumption and composition of unrecorded alcohol, but the report includes very little information on production in Asia [22].

### Summary of 2009 studies

In 2009, Pham Xuan Da published a series of eight papers that described traditional alcohol production in 83 sites in 15 provinces/cities in Vietnam and included a limited chemical analysis of 83 samples of traditional alcohol [23-30]. The average ABV of the alcohol samples collected was 35.5% +/- 6.8%. Almost all of the samples (90.4%) met acceptable standards for methanol with the highest exception being 4.7 times the established standard. None of the samples included heavy metals (Cu, Zn, Pb) above the accepted standards [23]. These alcohols were produced in small operations, 66.3% had two or fewer workers. Most of the operators had considerable experience, with only 28.9% having less than 10 years’ of experience making alcohol. The majority (89.2%) had learned their craft from their families, and 94.4% used family labour in their production [24]. Raw materials used were ordinary rice (50.6%), glutinous rice (39.8%) and other products like corn, sweet potatoes and cassava (9.6%). The use of non-rice products was more common in the north (17.9%) than the central (7.4%) or the southern region (3.6%). The fermenting yeast was typically purchased in stores (66.3%) rather than being made by the alcohol makers (33.7%). Buying yeast from local stores was more common in the south (81.5%) than the central (75.0%) or the north (42.9%). The point of sale was typically in the village where the alcohol was made (69.9%) or nearby (24.1%) with only 6.0% being sold beyond the local area. [25] The majority (88%) of these operations did not have any evidence of meeting established food safety standards. About 85.5% of the operations did have basic equipment for the sterilization processes, but 95.2% of the operators had no training in food hygiene and safety [26]. Of the owners 57.8% were male and 42.2% female. This relationship differed from north to south. In the North ownership was 50% male and 50% female, in the central region, 64.3% males and 35.7% female in the South 59.3% male and 40.7% female [27].

## Method

**Figure 1:**
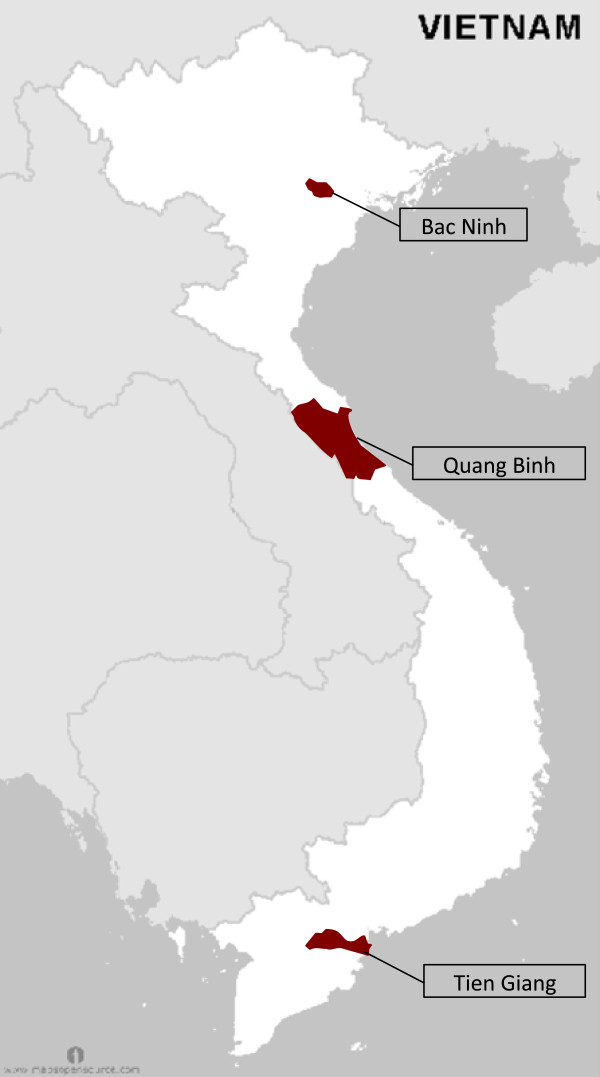
**Provinces selected for study in the northern Red River Delta region, the central Coastal Region, and the southern Mekong River Delta.** source: http://www.mapsopensource.com/vietnam-outline-map-black-and-white.html.

There are three distinct regions of Vietnam: the Red River Delta in the north, the narrow Central Coastal Region, and the Mekong River Delta in the south. In each region one province was selected as the primary sample unit. Provinces were selected to meet two criteria: The first criterion was that the majority of the population be Kinh people, the majority ethnic group of Vietnam. The second criterion was that the province be of average economic development, based on the province’s average per capita income and the growth rate of the per capita GDP. The rationale for the first criterion was to obtain information on alcohol traditions among the majority population, and save the study of alcohol traditions and practices among ethnic minorities for a later study. The rationale for the second criterion was to gather information that would be more representative of the country as a whole. The provinces selected were Bac Ninh, Quang Binh, and Tien Giang (Figure 1).

Using the same two criteria, and in consultation with local leaders, two districts were selected in each province, one commune was selected from each district, and one village was selected from each commune (Table 1).

**Table 1:** Areas selected for research

***Province***	**Bac Ninh**		**Quang Binh**		**Tien Giang**	
***District***	Yen Phong	Tu Son	Quang Ninh	Quang Trach	Cho Gao	Chau Thanh
***Commune***	Tam Da	Đong Nguyen	Vo Ninh	Quang Long	Hoà Đị;nh	Tan Huong
***Village***	Dai Lam	Quarter 4	Trung	Thuy Son	My Thanh	Tan Hoa

The following descriptive information was provided to the researchers by the village leaders from official government reports [no reference available].

Bac Ninh Province is in the northern Red River Delta region, northeast of Hanoi. Employment is 64% industry and construction, 24% trade and service, and 11% agriculture. In the Yen Phong district, rice cultivation is being replaced by vegetable farming and modern poultry and livestock production. The Tu Son district is a new satellite area of Hanoi, and is known for its iron forges, carpentry, and wood carving.

Quang Binh Province is in the narrow central coastal region that is squeezed between the ocean and the border with Laos. The economy of Quang Ninh district is 46% agriculture, forestry and aquatic production, 27% industry and construction, and 27% trade and service. Quang Trach district is 63% agriculture, 16% handicrafts, 13% forestry, and 7% aquatic production.

Tien Giang Province is a southern province on the Mekong River Delta about 70 km southwest of Ho Chi Minh City. In the Cho Gao district, agriculture and aquaculture accounts for 58% of the economy, industry and construction for 19%, and service and trade for 23%. The main products are rice, vegetables, coconut, cocoa, and dragon fruit. Chua Thanh is a developing industrial zone with newly developed industry accounting for 46% of the economy, agriculture for 32%, and service for 22%. Agriculture products produced in this district are rice, aquatic, fruits and livestock.

All the villages where this study took place were rural.

Within each village, three groups of men (Group 1: men age 30 and younger, Group 2: men age 31 – 50, and Group 3: men age 51 and older) and one group of women (of all ages) met for group interviews. The rationale for the gender distribution was that fewer women than men were drinkers and also reports that when woman did drink they drank less than men. In total, there were 24 villager group interviews that included, on average, 10 participants each.

At each commune, a separate interview group comprised leaders of the various agencies represented on the Commune People’s Committee, for a total of 60 Commune People’s Committee members interviewed for this study.

Lastly, researchers talked to the leader of each village after the completion of the group interviews. The interviews with village leaders provided local context and sometimes clarification of the information gathered in the group interviews. The village leaders often also provided additional information about the local traditional alcohols and their use. Researchers spent 2-3 days on average in each village.

The data were gathered by two Vietnamese teams, each consisting of a lead scientist and one assistant. The interviews were audio recorded and occurred in common village meeting places and typically lasted two or more hours. The assistant kept notes and occasionally prompted the interview. The third author was an observer who did not participate in the interviews.

Interviews and group discussions proceeded according to an outline of general, open-ended questions about local traditional alcohol (Table 2), with follow-up questions seeking clarification and expansion on the information contained in the replies of the interview group members to the initial questions. The aim was to gather information the participants themselves wished to supply on the topic of traditional alcohol.

**Table 2:** General questions used by researchers to guide discussions

**Group**	**General questions**
Commune members	Describe for me the local alcohol situation.
	Why do people make traditional alcohol?
	Why do people use traditional alcohol?
	How is traditional alcohol used?
	What risks are involved in traditional alcohol?
	What do you think about the production and use of traditional alcohol?
Commune Peoples’ Committee members	Why do people make traditional alcohol?
	Why do people use traditional alcohol?
	How is traditional alcohol made?
	What is the social and economic impact of traditional alcohol use?
	What are the local policies and attitudes related to traditional alcohol?
	How do you suggest traditional alcohol be managed?
Village leaders	Same questions as Commune Peoples’ Committee members

The focus of questions was production and use of the traditional distilled rice alcohol. There are a variety of traditional fermented and distilled beverages made in rural Vietnam that range from low to high alcohol-by-volume (ABV). The distilled rice alcohol was chosen for this study because it is both common and also it has a higher ABV, and thus may pose more health risks.

In most of the villages, in addition to interviews, the researchers were invited to visit local production sites and talk with the local producers. The description of the traditional method of production combines information from the interviews and from these observations.

The audio recordings and the field notes constituted the database upon which this report is based. There was no available software for analysis of transcripts in the Vietnamese language, so the analysis was completed manually. At the end of each day the research group met and identified patterns of information about traditional alcohol that were emphasized to them by the interview group members and then formulated questions for further exploration the following day. As the research progressed, themes developed, were confirmed and were subsequently enriched with new data. At the end of the process, the teams reviewed the project’s total findings and drafted an initial report, which was reviewed by the third author. This report was then revised and completed.

This research was approved by the scientific and ethics review committee of the Institute of Population and Social Studies, National Economics University of Vietnam, on 20 July 2011 (175/QĐ–VDS-HĐKH). All participants were adults. All participants signed a written consent agreement prior to their involvement.

## Results

In this section we present a summary of the findings from the fieldwork, and then in the discussion we consider the significance of these findings for policy deliberations intended to reduce alcohol-related risks and harms.

### Traditional method of production

The easy availability of rice dictated that practically all distilled spirits were made from one of three types of rice: regular rice, glutinous rice, or broken (cracked) rice. The local belief was that both the broken and the glutinous rice produced better quality alcohol. Broken rice is the cheapest and is, therefore, the rice of preference for alcohol making. The rice was heated over steam or boiled and then spread out on a flat surface to cool. When it reached the appropriate temperature, yeast was sprinkled over the rice and mixed well. The grain was covered and allowed to stand for 24 hours. Next, the fermented rice was placed in lidded buckets (typically plastic) and held for about 10 days. The buckets were filled with water and incubated for another 2 to 3 days. When the rice floated to the surface of the water the alcohol was said to be ready to distill.

The rice was then reheated in a covered pot and the steam ducted away to a water-cooled condenser, thus separating the alcohol. The first alcohol produced by this process was typically too strong (ABV 60-70%) and not considered good flavour—the distiller either discarded it or diluted it with alcohol of lower ABV. The second alcohol was lower ABV and better flavour. As distillation continued, the strength of the alcohol declined. The distiller decided when to discontinue the process. The entire process took several hours. Ten kilograms of rice typically yielded 6–8 litres of 45% alcohol or 10–12 litres of 30% alcohol.

The production of traditional alcohol did not differ markedly from north to south; however, in the south, in Tien Giang, the process differed slightly. After the rice was cooked and spread to cool, the yeast was mixed with water in a container and then the cooled rice was added. This is thought to allow the rice to absorb the yeast evenly. The rice was held around 37-38 degrees C during the fermentation process for approximately 10 days. The distillation process was the same as described earlier.

Over time some changes have occurred in the materials used for making traditional alcohol. A government official in private conversation said…. “*If all traditional alcohol was produced using traditional products* [wood and bamboo] *we would not have any new dangers from the introduction of metal* [copper and lead] *piping and plastic containers”.*

### The yeast

The fermentation of grains is a natural process requiring no human intervention as long as the appropriate yeasts are present. However, for reliable production the yeast selected for the fermentation process is important. Alcohol makers will tell you that the quality of the yeast determines the quality of the alcohol’s flavour, aroma and taste.

The communities studied for this project described local preferences for the yeast used in making rice spirits. In Bac Ninh, in the north, the residents described a special combination of herbs mixed with the yeast called Men Bac (Chinese herbal medicine). The mixing followed a secret family recipe known only to the yeast makers and passed on to the next generation when they were near death. As one family member said:

Making Men Bac is hereditary. It may contain 50 to 60 herbal medicines. The herbal medicines are dried, cleaned, melted and crushed with rice and then mixed together with the yeast. The ratio of mixing the ingredients determines how good the yeast. Only I know. As of now I have not passed this information on to anyone else. (Focus group discussion (FGD) female, Bac Ninh)

In Quang Binh, again, according to secret family recipes, yeast was often made with galangal, a type of ginger.

“Galangal for yeast making must be planted on hills and aged for 3 years or more…. Only those households whose families know the recipes know exactly how much is mixed with the yeast and how much (yeast) should be mixed with 10 gallons of rice. (Male, 31 -50 years, Quang Binh).

Alcohol made with galangal yeast was said to be of higher-quality, with higher ABV, and could be consumed to the point of drunkenness without a hangover headache.

In the south, alcohol was mostly fermented with pre-packaged yeast available in the market, either made locally or imported from the north or from China. The locally-made pre-packaged yeast includes instructions printed on the package, but most of the village residents said the instructions are ignored and local practice dictates the procedure. Residents said that Chinese yeast could be used to ferment rice without first cooking the rice and that fermentation time was shorter (a good thing) but the alcohol produced was “less delicious.” From the interview data, however, it was difficult to discern the perceived differences between yeast supposedly imported from China and local marketplace yeast. Chinese-made yeast was regularly blamed for poor quality alcohol. People said that alcohol made with Chinese yeast contained toxins, caused poisonings, and left drinkers with headaches and other discomforts.

### The durability of tradition

Throughout the interviews there were frequent references to alcohol making as representing an important tradition. This reference to traditions was most often invoked when interviews turned to past or present attempts to control and/or reduce the manufacture of traditional alcohol, and when the virtues of traditional alcohol were discussed. In this context it was often suggested that the taste represented the tradition, such that the unique taste of traditional alcohol was part of its appeal and also contributed to local and generational pride in its production.

Older participants recalled historic attempts to control traditional alcohol making, and recounted stories like the following:

In the French period it (alcohol making) was prohibited and the equipment was confiscated and the alcohol maker fined. In the 60s and 70s the government prohibited alcohol making and officials were sent to capture alcohol making equipment. Local people hid them in the ponds. Even with the penalties alcohol making continued. It was a tradition. (Focus group interview with CPC leaders, Bac Ninh)

### Drinking patterns and reasons for drinking

Data from the interviews indicated that the custom in most places was to drink traditional alcohol daily, usually during lunch and the evening meal, at home, and sometimes in the evenings after dinner. Older people may drink a little in the morning. Typically no more than 2-4 cups were consumed on an occasion. Men drank more alcohol, in terms of both quantity and frequency, than women. There was more drinking on the weekends than during the week and more drinking when the weather was cool. Beer was the alcohol of choice in hot weather when the men were busy in the fields. People in the central and south regions tended to drink more than people in the north. Beer was increasingly the beverage of choice among younger people.

Drinking was both a daily activity, much like eating, and it was also a special activity associated with special events. At festivals in January and February there was a lot of drinking. As one Bac Ninh village leader phrased it, “Alcohol and dog meat are essential at village festivals.” The amount of alcohol consumed depended upon the occasion. Festivals and weddings were happy events and were accompanied by more drinking than sad events like funerals and anniversaries for the dead.

Reasons most often given for drinking traditional alcohol were to improve digestion, make meals more delicious, increase happy feelings, and express special relationships. People described regularly taking medicinal alcohol—that is, traditional alcohol mixed with prescribed herbs, insects, or animal parts. Alcohol is routinely offered to guests as a traditional show of hospitality. *“Guests would consider themselves unappreciated if they were not invited to a cup of alcohol”* (Male, 31–50 years, Quang Binh). Alcohol, especially commercial/labelled alcohol, was given as a gift, especially at Tet (New Year). Expensive commercial alcohol was typically not drunk, but displayed.

### Price

Sellers based price on quality, and quality was determined by per cent of alcohol by volume, the raw material used, and the flavour. The price also varied by region and commune.

In Bac Ninh and Tien Giang alcohol made from ordinary rice with a concentration of about 30% cost 15,000 Vietnam Dong per litre (VND/L) (≈.52 EUR). Alcohol made from ordinary rice with concentrations 45 to 50% cost 25,000 – 30,000 VND/L (≈.87–1.04 EUR). Alcohol made from glutinous rice cost 40,000 to 50,000 VND/L (≈1.30–1.74 EUR). Alcohol made from broken rice cost 30,000 VND/L.

In Quang Binh there was a difference in the price of the alcohol made in the Vo Ninh commune and alcohol made in the Quang Phong commune. In Vo Ninh commune alcohol made from ordinary rice sold for 20,000 VND/L while alcohol in Quang Phong sold for 40,000 VND/L. Alcohol made from glutinous rice in Vo Ninh sold at 24,000 VND/L compared to 16,000 VND/L in Quang Phong. In Vo Ninh alcohol made from glutinous rice and sold with the commune label cost 70,000 – 80,000 VND/L(≈2.43–2.78 EUR). Vo Ninh has a long-standing reputation for its alcohol and has a successful commune alcohol cooperative.

For comparison, Vietnam-made commercial beer cost 45,000 VND/L, imported Heineken beer cost 48,000–75,000 VND/L, and Coca Cola cost 39,000 VND/L.

### Distribution of traditional alcohol products

This study found four distribution patterns. The basic pattern of traditional alcohol distribution involved the family that made the alcohol consuming most of it themselves and giving the rest away to close relatives and neighbours. This was truly “informal” or “non-commercial alcohol”, inasmuch as there was no selling and/or trading. A few families did sell or trade some of their surplus to their neighbours.

The next pattern of distribution involved a single small manufacturer both producing and selling or trading alcohol directly from the manufacture location. Customers were members of a social network of individuals known to the manufacturer. These were repeated sales to individuals on a daily, weekly or less regular basis. These manufacturers were sometimes asked to supply larger amounts for special functions like weddings and housewarmings. Larger manufacturers might supply local restaurants or stores in close proximity. At this level of distribution, the manufacturers and the sellers were typically well known to one another.

The third pattern was distribution via cooperative. In the central region (Quang Binh) alcohol was often distributed through alcohol-making communes. In these communes, the village cooperative purchased the locally produced alcohol from cooperative members and mixed and possibly further refined the alcohol, bottled, labelled and then distributed it through an established sales network. In the south (Tian Giang), locally-made alcohol was distributed at kiosks throughout the town.

The fourth pattern of distribution was the largest scale production of traditional alcohol. These producers distributed their alcohol to more distant locations and larger cities, selling directly to specific sites for resale. Some of these larger scale operations sold their alcohol to an agent who then resold it in other locations.

### Perceived benefits of traditional alcohol

When asked to describe the reasons why traditional alcohol was important, participants referred to the importance of preserving long-standing traditions, such as the traditional alcohols that have been made for as long as anyone could remember. Traditional alcohol is an important part of local seasonal celebrations and rites of passage and is prized because it is a local product and because it satisfies local values and tastes. Also frequently mentioned was the economic role alcohol manufacture gives women in the household and the community, when there were few other opportunities. Alcohol making is home-based and the work hours are flexible, allowing women to continue to attend to child rearing and housekeeping while contributing to the family income.

The spent grain from alcohol making was fed to livestock, contributing a second important source of family income and local economy. People believed that grain that had been used to make alcohol was a more nutritious animal feed. Whether this is true or not, the grain certainly served two functions, supporting both alcohol making and livestock production.

Larger scale alcohol manufacturing contributed to the local economy by providing jobs to a few local people, and the profits from sales outside the village generated income for the local community.

Participants believed that locally-made alcohol was good quality, satisfied local tastes, and was safe compared to alcohol coming from outside the local area, including commercial brands of alcohol. Traditional alcohol was also seen by the people interviewed for this study as having many observable idiosyncratic social and health benefits. One woman, who laments the fact that she does not make her own alcohol, explains it this way:

When he [her husband] meets someone he always stops and has a drink. Sometimes he is away from home for two or three days just for drinking. I should make alcohol for him then at least he can drink at home. If he is drunk at home I can take care of him. (Female, TianGiang)

And a young person reported:

Alcohol has its own benefits if we drink regularly in small amounts. For example for old people who drink alcohol with herbs it makes them stronger. (Male, age 30 or younger, Bac Ninh)

And an old man stated:

Drinking a cup of alcohol to keep warm is good. A cup helps avoid catching a cold at night. (Male, age 51 or older, Quang Binh).

### Perceived risks of traditional alcohol

Most of the people involved in these interviews could not think of any widespread negative effects from traditional alcohol. Some mentioned that the wood fires burning during the production process polluted the air. Others countered this describing the benefits of alcohol making facilities being near sources of fuel. For example, an alcohol production facility close to a furniture making operation could use the sawdust and waste wood for making alcohol. A few of the villagers raised the issue that large-scale alcohol production led to the raising of a large number of animals for consuming the used grain, and the waste from so many confined animals caused water pollution and bad odours—this, however, was not a widely expressed sentiment. Also not frequently mentioned as bad outcomes were traffic crashes, bad public behaviour, and family problems, all connected with excessive drinking and alcohol abuse.

Some drunken people at festival speak loud, shout and hit each other… sometimes hit their wives, behave badly to their dogs (Community leaders, Bac Ninh)

Some run their bikes less speedily… Sometimes hit their wives… A few cases of crimes committed… Sometimes cause social disorder… (People’s committee member leader Bac Ninh)

Wife doesn’t give money to husbands to drink alcohol and even complain so those husbands are annoyed and hit their wives (male, age 31–50, Tian Giang)

Man is hot tempered, especially with wine in the body … If wife complains instead of being patient conflict will occur (male, age 51 or older, Tien Giang).

### Declining production?

Participants made frequent mention of declining production of traditional alcohol. Their explanations for the decline varied. Perhaps the most obvious was the change in the alcohol marketplace. There is now a greater availability, even in the rural areas, of a wide range of alcohols, beers, wines and spirits. Prices continue to fall and availability to increase.

The traditional pattern is further changed by the increasing number of young people who are leaving the areas where traditional alcohol is made and used to go live in urban areas where opportunities to work in non-agricultural jobs has increased their incomes, expanded their purchasing power, and expanded their knowledge and experience of the wide range of available commercial alcohol products.Associated with this increased access is also a perception that the use of imported and branded products carries a particular message of status, a belief that is more common among young people.

Meanwhile in the rural areas there is now less opportunity for raising livestock. As production and agricultural methods have become more diversified and westernized, rural land for agriculture, especially close to the city, is getting scarce and increasing in price, making agricultural uses less profitable. The availability of chemical fertilizer has decreased the demand for animal produced fertilizer.

There is also a concern about the declining safety and quality of the yeast available to make traditional alcohol and about the safety of traditional alcohols in general. Today, the occasional rumour of poisoning from traditional alcohol circulates quickly.

All of these changes suggest traditional alcohol production in Vietnam may be declining, but interviews with village leaders and with members of the local Commune Peoples’ Committees suggest that the situation is more nuanced. In Bac Ninh, in the north, both of the sampled communes, Tam Da and Dong Nguyen, had long-standing reputations for making fine quality alcohol, and yet in Tam Da the Commune officials estimated the proportion of households making alcohol had declined from 90% to around 30% in the last 5-10 years. Officially, the Tam Da Commune Peoples’ Council and the Peoples’ Committee discourage alcohol production because of its negative consequences, which the leaders listed as social discord among family members and arguing and fighting among community members.

Yet, in Dong Nguyen, in some central villages and towns, the number of alcohol producing households is around 70 to 80% and thought to be increasing because rice is plentiful and the commune’s official position is to develop homemade alcohol production as a traditional village craft.

In Vo Ninh (a commune in Quang Binh Province in the Central region) the commune has officially adopted a resolution to develop traditional alcohol making as a village craft. In this commune an alcohol cooperative was formed and small makers pooled their daily production for further refinement, bottling, and labeling. Total daily production was between 5,000 to 7,000 litres. The commune then managed the sale and distribution of the alcohol. The cooperative started in 2006 with 25 permanent household members and now has about 100 members, some producing as little as 5 litres a day. An Executive Board is responsible for obtaining/providing the rice and the yeast and ensuring a quality finished product. The member households were expected to produce a stable amount of alcohol. Their cooperative-labelled product has become popular, and the profits from alcohol sales along with the profits from the livestock raising program that uses the spent grains have allowed the commune to invest in new technologies and equipment to support its businesses (Commune leader, Quang Binh).

This situation contrasts with the second Quang Binh commune, Quang Long, which has no alcohol policy, where it is reported that in the past as many as 100% of the households produced alcohol. Today it is less than 25%, with alcohol typically made in only three or four villages (Male, 31-50 Quang Binh).

In Tien Giang, in the south, alcohol making is less common than in the central or northern regions. In neither of the sampled communes was alcohol making a major endeavour. The leaders in one commune did not want to develop alcohol, while the leaders at the other commune wanted to register their brand and promote alcohol as a traditional village craft, but they had not made much progress.

## Discussion

In this section we discuss each of the findings described earlier and suggest their significance for policy considerations designed to reduce alcohol-related risks and harms.

### Traditional methods of production

Interviews and observation confirmed that the methods of traditional alcohol production are simple and do not depend upon any sophisticated technology. It is likely these are the same methods that have been in practice for hundreds of years. With the exception of the recipes for special yeasts, which in some cases are closely guarded family secrets, knowledge of how to make alcohol is readily available and the process is cost-effective, requiring only fire and materials for distillation.

So straightforward is the process that in some villages families rotate the responsibility to produce alcohol. One family will produce it for a period of time, usually several years, and then another family assumes the responsibility (Male, age 30 or younger, Tian Giang).

### Yeast

Appropriate yeasts are essential for the making of alcohol, but how the other ingredients added to the yeast affect the final product is a matter of conjecture. A systematic analysis is needed of the range of yeasts made and used and purchased in the market. The village residents used the topic of yeast as a convenient vehicle to express anti-Chinese feelings.

### Durability of tradition

The alcohol studied here is best called Vietnamese (Kinh) “traditional” alcohol. It is not illicit. In some communes, alcohol manufacture was “informally” made for home consumption, but in other communes it was formalized as a local cooperative industry. The distilled alcohol was made to meet the tastes of the local people and depended on locally available ingredients. Imported ingredients (imported yeast, for example) were considered inferior. Changes over time have occurred in the type of container (plastic substituted for pottery, metal pipe substituted for bamboo) but not in the recipe or procedure. Traditional practices, such as pouring alcohol on the graves of deceased relatives and offering alcohol to divine spirits suggests that alcohol in this culture has values unrelated to its chemical effects on the body or its value to the local economy. Thus any policy to change alcohol availability will impact more aspects of the society than its health.

The tradition of alcohol making in Vietnam has resisted at least two attempts in the Twentieth Century to regulate or prohibit it. Peters [31] describes the French colonial government’s attempts, between 1902 and 1913 to first tax traditional alcohol and then establish an alcohol monopoly to reduce the making of traditional alcohol. These attempts failed and, in the process, reduced the living standards in many villages, created health problems with their “modern” technologies, increased grassroots anti-colonialism, and attracted negative press in the papers of Europe. The inability of the French colonial government to recognize local traditions related to beverages and taste is ironic because the French themselves have sophisticated traditions based on the taste of both food and wine. They simply could not recognize or appreciate this in others.

In the 60’s and 70’s, further attempts were made to regulate Vietnamese traditional alcohol production. These also failed as the government officials underestimated the degree to which local people valued traditional alcohol and were willing to take the trouble to hide alcohol making equipment in ponds and risk imprisonment.

Beginning last year, a new Decree, 94/2012, requires all those who manufacture traditional alcohol to register with the local authorities. Exactly how this decree will affect production remains to be seen. Initial scepticism of any real effect is based on the amount of time it will take for knowledge of the new decree to reach the rural areas, whether or not local people will consider it important enough to comply, and how diligent local officials will be in enforcing the decrees requirement [32].

Since there does not seem to be much point in making another attempt to prohibit the manufacture or sale of traditional alcohol, one way to proceed may be to gather information on the risks and harms, then develop appropriate programs to reduce existing risks without creating new risks and harms. Commune-based production appears to be one such approach where small production units are possible and the shared social responsibility for quality is maintained. This point is made in more detail below in the discussion of distribution methods.

### Drinking patterns and reasons for drinking

Participants described drinking one to two cups at the end of the day, drinking with meals to improve digestion, and drinking with meals to enhance the flavour of food. Drinking with meals is generally a less detrimental pattern of alcohol use, as is drinking at home. This study did not try to assess quantity of pure alcohol consumed as the strength (ABV) of the alcohol varies as does the size of the cups used. However, daily quantities for men were typically reported as 2 – 4 cups and less for woman. Around two to four cups a day appeared to be considered typical drinking by the study participants. Two to four cups per day results in a relatively high average per capita consumption.

Episodic heavy drinking as well as intoxication occurred at weddings and festivals, occasions for which more alcohol than usual is provided. Reports about festival occasions suggest 5 or more being consumed by men. The risks from these occasions would depend on the number of such occasions during the year and, most obviously, the mode of transportation from the event. The risks arising from festive drinking events may be moderated by the mix of genders and the mix of ages of the participants, and also by whether the event was happy or sad. Happy situations were associated with more drinking, as were all-male drinking situations, and situations involving only young men. Vietnamese cultural norms that are likely to moderate bad behaviour include: women drinking less frequently and drinking less volume than men, the presence of extensive family relations in the village, a concern for the reputation of the family, and filial attendance to parental warnings.

These data did not contain descriptions of indigenous cultural controls on the individual use of traditional alcohol and the acceptability of different behaviours after drinking. In previous research in Thailand “rules” surrounding drinking and drunkenness were so culturally integrated into the social fabric of the community that participants felt no need to mention “the obvious” during interviews [33]. There are hints in the data here that suggest the existence of “rules” in Vietnam culture– for example, more drinking at happy events, less drinking at sad events, males drinking more than females, exceptional drinking patterns at festivals. These cultural controls deserve identification and study so they can be reinforced to reduce alcohol-related risks and incorporated into future efforts with the same objective. Basic fieldwork of the type illustrated in this study will be needed to identify these important indigenous social values and behaviours. Room [34] and other have suggested the possible role of indigenous values and practices in reducing alcohol related risks, but the idea has not generated much interest, maybe because these practices vary greatly among different societies [34-36].

If indeed indigenous values and practices surrounding the use of traditional types of alcohol do contribute to reduced alcohol-related risk then an expanding commercial alcohol market could actually increase risk by decoupling alcohol beverages from a traditional context. Participants described how traditional alcohol is made, sold, and consumed within a confined cultural context: the traditional alcohol is tightly linked to traditional schemas about gender, and about how much, how frequently, where, when, and with whom one traditionally uses this traditional drink. In other words, Vietnamese traditional alcohol comes with Vietnamese traditional assumptions that curb its use and lower the risk of harm. If the Vietnamese traditional alcohol is replaced with introduced commercial types of alcohol, it is important to consider that this introduction comes without a cultural context, traditional constraints, or social agreements about acceptable use. As a consequence, users would have carte blanche in their patterns of use. Lacking a long-standing cultural context to curb use, then alcohol use will have to be limited by laws and enforcement. This is a much more expensive way to prevent overindulgence and reduce risks and harms, but may become necessary if younger people adopt tastes for non-traditional (commercial) drinks and ways of drinking.

Future surveys that are designed to measure frequency and/or quantity of alcohol use in this region will have to be carefully worded to count the medicinal cup of alcohol, the after-work cup, the cup with meals, the cup drunk with the visitor, since participants often do not themselves define all these situations as “drinking”. This is especially true for woman. And, of course, there is the problem of not counting the bottle of alcohol that has been bought and displayed, not drunk.

### Price

The participants described the strength of traditional alcohol between 30% and 50% alcohol-by-volume. This finding is in line with Pham Xuan Da’s chemical analysis [23]. The price being charged for the alcohol by sellers in this study is inexpensive and affordable. Vietnam’s gross national income (GNI) per capita is listed by the World Bank as 1550 (current US$) and projected to grow. The inexpensive price carries a risk associated with easy accessibility. The existing traditional pricing structure described in this data—where higher ABV beverages are sold at a higher price—is another example of a traditional practice that lowers risk. Raising prices through taxes carries with it the possibility of reducing the number of small alcohol producers whose distribution system, described in the next section, also contributes to the reduction of risk. At the same time reducing the number of small alcohol producers decreases the number of families who can meet the costs of education and health care for their children. As a community leader in Quang Binh said:

*One advantage of making* [traditional] *alcohol at home is to have extra income to send children to school. The second advantage is that it finds jobs for women 30 to 40 years of age and you can also use the wine dregs to raise pigs* [another added value].

### Distribution patterns

Three of the distribution patterns described by participants appeared to reduce some of the risks associated with alcohol manufacture, sale, and consumption: 1) manufacture for use by family and friends, 2) manufacture for sale to local individuals and businesses, and 3) communal alcohol manufacture and sale to established customers. At these levels, consumers and manufacturers are well known to one another and have traded with one another repeatedly. This knowledge and experience prevents intentional contamination of alcohol products. Knowledge and experience likely has eliminated the manufacturers who sold poorly made, unintentionally contaminated, or dangerous alcohol in the past. In other words, the situation in rural Vietnam has built-in safeguards against some of the risks of illicit or informal alcohol that have been documented in other parts of the world. Attempts to introduce price and quality controls on this type of alcohol, by a centralized agency for example, should be careful to leave in place the safeguards that are present in the current traditional distribution systems.

Traditional alcohol recipes and manufacturing methods are prized family assets. Local alcohol makers are respected for their skills and the quality of their product and it is unlikely they would knowingly do anything to destroy their reputation. As one woman in an interview group said*…. “If you drink alcohol made in your own village you cannot be poisoned* (Woman, Bac Ninh).

Similarly, many of the people we interviewed preferred the local alcohol because they did not trust the purity of commercial alcohols.

The fourth type of distribution found in this study, however, reached beyond the social network and the safeguards inherent in it. Selling to distant locations, as well as the involvement of middlemen (wholesalers) increases the risk of diluting, adulterating, and contaminating traditional alcohol. These larger distribution networks can be influenced more by profit and are not constrained by close relationships to customers. Participants reported that alcohol distributed by agents was of lower quality, made from poorer quality products, possibly adulterated with industrial alcohol or diluted with water. They also believed that agents mixed alcohols from different producers. Nevertheless, there is a market for this alcohol, usually because of its lower price and especially in urban areas, where it is purchased by workers who have come from the rural areas. Ironically, the manufacture and distribution of *commercial* alcohol in Vietnam falls into the fourth category of distribution system—i.e. higher risk distribution system.

Now and then one encounters traditional alcohol being sold from a stand on the side of the road. This practice increases the risk for drink driving. At this time the lack of high-speed roads and scarcity of vehicles larger than a motorcycle limits harms from drink driving, but this is a situation where carefully implemented education and traffic enforcement programs could reduce present and future risks and could prepare people to support the enforcement of anti-drink-driving regulations.

### Perceived benefits

Participants listed a number of benefits from traditional alcohol as they saw them: the economic of benefits from alcohol manufacture and sale, local jobs, income for women, livestock production, and communal income. They liked traditional alcohol for its cultural heritage, quality, flavour and safety. The social benefits included family relationships (*drinking at home*), religious, festive and celebratory drinking. Personal health benefits included relieving fatigue, keeping warm, aiding digestion, enhancing food, and—when mixed with herbs—treating and/or preventing illnesses. Policies intended to reduce risk and harm associated with traditional alcohol will have to carefully consider how people regard its benefits.

### Perceived risks

Our findings from the recorded interviews and discussions, as far as how local people perceived risks, did not describe alcohol-related health risks as we would envisage them from a western perspective. The villagers described effects on the environment mainly, and they linked excessive drinking to traffic crashes, public bad behaviour and fighting, and family problems. Overall, it appeared that local people perceived few risks connected to traditional alcohol production and use. More closely detailed questioning will be needed to discover the real extent of any of the problems mentioned by the villagers and what causes the difference between well-behaved drinkers and badly behaved drinkers—what types of alcohol are consumed, how often, how much, and in what circumstances.

In other regions of the world, poisoning by illicit and informal alcohol is reported as an on-going problem. In this Vietnam study, not one of the more than 300 people interviewed cited a specific poisoning case known to them personally. Poisonings do occur and these events make the print and electronic news, which likely magnifies the perception of their likelihood and contributes to the frequency of the retelling of the event. Lachenmeier and colleagues [37] in a pilot study of Vietnam samples of “homemade (artisanally manufactured and unrecorded)” reported no “toxicologically relevant substances”. They concluded that “… except for ethanol, no compound that may lead to acute toxic effects were detected”. Analyses so far have not uncovered risks, beyond those of ethanol. This would suggest that policies based on the perceived risk of poisoning may be unfounded and would not resonate well with the local population if used as a rationale for regulations.

### Declining production

The culture and economy of traditional alcohol appears to be in a state of change. In some areas, we found efforts to ensure the continued production of traditional alcohol, as in those communes that designated alcohol production as a traditional craft and managed its production and sale for the betterment of the whole community. In other areas, alcohol manufacturing was unappreciated as a significant tradition and even discouraged. At the same time we observed the beginning availability of commercial alcohols in small village shops.

As young people’s tastes change, the practice of making and drinking Vietnamese traditional alcohol may simply disappear on its own, and the possible risks associated with “informal” alcohol will go away with it. Young people may prefer a lower ABV alcohol beverage, such as beer, which could lead to lower harms from alcohol over time or it could lead to beer related problems common in some Western countries. Drinking larger amounts of lower ABV alcohols, especially in male only settings, may not be a net benefit from reduced use of traditional alcohols.

A few problems might be anticipated if young people switch to commercial brands, including in the rural areas. (1) Commercial alcohol distribution does not have the intimate social safeguard that minimizes the temptation to adulterate or dilute products. (2) Youth will face increased exposure to media marketing by the commercial alcohol industry that comes along with the introduction of commercial brands of alcohol. Traditional alcohol, by contrast, is “marketed” by local reputation or by word of mouth. (3) The risk of drink driving on the country’s roads rises, since the urbanized young people are travelling between city jobs and rural hometowns. Traditional alcohol was drunk in the home and in the village. (4) Young peoples’ work situations in the urban areas are typically in male only settings, again encouraging the use of commercial alcohols, making them less likely to drink traditional alcohol when they do return to their rural homes.

Interviews with Commune leaders and committee members demonstrated that there is already a level of local or collective control surrounding traditional alcohol in the communities we studied—this was true both when the collective promoted alcohol manufacture as a village craft, which brings alcohol makers into an organization that can oversee quality and discourage independent operators, and when the collective decided to discourage alcohol manufacture. The success of some communes in joining together to maintain the traditional family production units while sharing in commune controlled sales and distribution suggest some promise in maintaining the best traditional values associated with production and minimize the risks associated with wide distribution. The statement by the government official that alcohol should be produced “*using traditional products* [wood and bamboo]” suggests appreciation by leaders for the quality of the traditional alcohol. In fact, the official’s statement suggests that a project to urge the return of the crafting of local traditional alcohol to its original processes and materials might be both a sound and culturally acceptable policy that could lower risks.

## Conclusions

The information gathered in the 3 provinces in Vietnam suggested that the process for making traditional distilled spirit alcohol followed a long-standing tradition, and that the tradition withstood at least two attempts in the Twentieth Century by outside cultures to dismantle it. The distilled alcohol was made to meet the tastes of the local people, depended on locally available ingredients, and imported ingredients (for example, Chinese yeast) were considered inferior. Changes over time have occurred in the type of container (plastic substituted for pottery, metal pipe substituted for bamboo) but not in the recipe or procedure.

The open, legal, integrated and long-standing nature of traditional alcohol manufacture in rural Vietnam means that moves to regulate or tax this type of alcohol could easily trigger the formation of underground manufacture and black market distribution—both prospects that would introduce new risks into the situation.

These findings from rural Vietnam should not be generalized to traditional alcohol production in other countries, particularly those in Africa, Eastern Europe, and Latin America where there is evidence of clearly dangerous contaminants and links to organized crime [3]. It is important to carefully study traditional alcohol in the Asian cultural context, where the excesses evident in Africa, Eastern Europe, and Latin America are not evident. Therefore WHO’s global strategy notes that one of the “guiding principles” to reducing harms from alcohol is considering the national, religious, and cultural context in recommending actions (page 9 and page 34) [1].

There is little pre-existing knowledge and understanding of the traditional alcohol making process, role, benefits, and risks for Vietnam. This ethnographic study provides new knowledge and insights for policy planning and provides guidance in developing questions for qualitative and quantitative studies to gather data upon which to build sensible public policies.

## Limitations of this study

This paper describes traditional alcohol making and use in three provinces in Vietnam. The information was gathered from direct conversations with local people in the three rural areas. This paper is based on a description of traditional alcohol manufacturing, distribution and use as described by the individuals interviewed. This was an ethnographic study, so the results are not based on a random sample of subjects nor do the results describe a comprehensive view of Vietnamese traditional alcohol. Nevertheless, there was a reasonable concurrence between the descriptive data reported in the previous studies by Pham Xuan Da [23-30] and the results obtained in these interviews, suggesting a degree of face validity.

## Competing interests

The fieldwork upon which this paper is based was supported by a grant to Luu Bich Ngoc from the International Centre for Alcohol Policies (ICAP). Luu Bich Ngoc has received funds from ICAP for other projects and for travel to international meetings. She also receives support from the Institute for Population and Social Studies, National Economics University, Hanoi – Vietnam.

Ian Newman’s involvement in the fieldwork was supported by ICAP. He serves as a member of an advisory committee to ICAP and has received funds from ICAP for other projects and to attend and speak at international meetings. The Nebraska Prevention Center for Alcohol and Drug Abuse, which he directs at the University of Nebraska-Lincoln, receives funds from the Nebraska Department of Roads, the US Department of Education, and the Nebraska Department of Health and Human Services. The American Exchange Center at Xian Jiaotong University, which he co-directs, receives funds from the US State Department, the University of Nebraska, and Xi’an Jiaotong University.

The writing of this paper was not funded by any agency. The ideas expressed in this paper and the conclusions stated are those of the authors, and do not represent the ideas of any agency or organization.

## Authors’ contributions

LGN and NTT collected the interview data. IMN designed the study and the data collection process and observed the data collection. All three authors analysed the data, interpreted the results, and contributed to writing this report. All authors read and approved the final manuscript.

## Pre-publication history

The pre-publication history for this paper can be accessed here:

http://www.biomedcentral.com/1471-2458/14/731/prepub
